# Trends in malaria epidemiological factors following the implementation of current control strategies in Dangassa, Mali

**DOI:** 10.1186/s12936-022-04058-0

**Published:** 2022-02-23

**Authors:** Mahamoudou Touré, Moussa Keita, Fousseyni Kané, Daouda Sanogo, Salim Kanté, Drissa Konaté, Ayouba Diarra, Nafomon Sogoba, Mamadou B. Coulibaly, Sekou F. Traoré, Michael Alifrangis, Mahamadou Diakité, Jeffrey G. Shaffer, Donald J. Krogstad, Seydou Doumbia

**Affiliations:** 1 West African International Center of Excellence for Malaria Research, Bamako, Mali; 2 Faculté de Médecine et d’Odonto-Stomatologie (FMOS), Université des Sciences, des Techniques et des Technologies de Bamako, Bamako, Mali; 3 Faculté de Pharmacie (FAPH), Université des Sciences, des Techniques et des Technologies de Bamako, Bamako, Mali; 4 Departments of Tropical Medicine and Biostatistics, Tulane School of Public Health and Tropical Medicine, New Orleans, LA USA; 5 Department of Immunology and Microbiology, Centre for Medical Parasitology, University of Copenhagen, Copenhagen, Denmark; 6 Department of Infectious Diseases, Copenhagen University Hospital, Copenhagen, Denmark

**Keywords:** *Plasmodium falciparum*, Malaria, Control strategies, Failure, Dangassa, Mali

## Abstract

**Background:**

Over the past decade, three strategies have reduced severe malaria cases and deaths in endemic regions of Africa, Asia and the Americas, specifically: (1) artemisinin-based combination therapy (ACT); (2) insecticide-treated bed nets (ITNs); and, (3) intermittent preventive treatment with sulfadoxine-pyrimethamine in pregnancy (IPTp). The rationale for this study was to examine communities in Dangassa, Mali where, in 2015, two additional control strategies were implemented: ITN universal coverage and seasonal malaria chemoprevention (SMC) among children under 5 years old.

**Methods:**

This was a prospective study based on a rolling longitudinal cohort of 1401 subjects participating in bi-annual smear surveys for the prevalence of asymptomatic *Plasmodium falciparum* infection and continuous surveillance for the incidence of human disease (uncomplicated malaria), performed in the years from 2012 to 2020. Entomological collections were performed to examine the intensity of transmission based on pyrethroid spray catches, human landing catches and enzyme-linked immunosorbent assay (ELISA) testing for circumsporozoite antigen.

**Results:**

A total of 1401 participants of all ages were enrolled in the study in 2012 after random sampling of households from the community census list. Prevalence of infection was extremely high in Dangassa, varying from 9.5 to 62.8% at the start of the rainy season and from 15.1 to 66.7% at the end of the rainy season. Likewise, the number of vectors per house, biting rates, sporozoites rates, and entomological inoculation rates (EIRs) were substantially greater in Dangassa.

**Discussion:**

The findings for this study are consistent with the progressive implementation of effective malaria control strategies in Dangassa. At baseline (2012–2014), prevalence of *P. falciparum* was above 60% followed by a significant year-to-year decease starting in 2015. Incidence of uncomplicated infection was greater among children  < 5 years old, while asymptomatic infection was more frequent among the 5–14 years old. A significant decrease in EIR was also observed from 2015 to 2020. Likewise, vector density, sporozoite rates, and EIRs decreased substantially during the study period.

**Conclusion:**

Efficient implementation of two main malaria prevention strategies in Dangassa substantially contribute to a reduction of both asymptomatic and symptomatic malaria from 2015 to 2020.

## Background

Despite intensive and continued implementation of evidence-based interventions, malaria remains a worldwide public health problem, particularly in sub-Saharan Africa (SSA). With a significant geographical and seasonal variation, SSA is the most affected region with respect to malaria-related deaths. Between 2000 and 2015, malaria cases declined from 238 to 218 million detected cases [1]. Malaria deaths decreased from 839,000 in 2000 to 409,000 in 2019 [1]. However, this dynamic has changed over the last 5 years, with 11 million more malaria cases in 2019 compared to 2015 [1].

During the past decade, Mali and most SSA countries started the progressive implementation of proven malaria prevention and control strategies, such as the distribution of insecticide-treated bed nets (ITNs), indoor residual spraying (IRS) in targeted geographic areas, improved access to healthcare with early diagnosis and prompt treatment, prevention and rapid management of malaria epidemics and surveillance, provision of intermittent preventive therapy to pregnant women (IPTp), and seasonal malaria chemoprevention (SMC) for children under 5 years old. The Malian Government and its partners have been offering free malaria diagnostics and treatment to children under 5 years old and pregnant women since 2008 [2–4].

Malaria epidemiological factors in Mali are associated with the four eco-climatic zones in the country: (1) the Sahara zone, with a short rainy season where epidemics of malaria could occur; (2) the Sahel zone, with mostly irrigated rice production enhancement projects where malaria epidemiology varies according to water used and agricultural activities; (3) the Sudanese-Guinean zone, where transmission can last up to 6 months a year; and, (4) the Inner Delta of the Niger River, where malaria transmission continues year-round.

In addition to IRS, which is implemented in only two of Mali’s districts, all other control and prevention strategies are present countrywide regardless of the differences in transmission dynamic. As in many malaria-endemic countries, one of the main gaps in control strategy planning and implementation is the lack of accurate data on the disease from local health facilities. Community-based cohort data on malaria prevalence and incidence over several consecutive years are more reliable and can provide evidence-based and area-specific interventions [5–8].

**Fig. 1:**
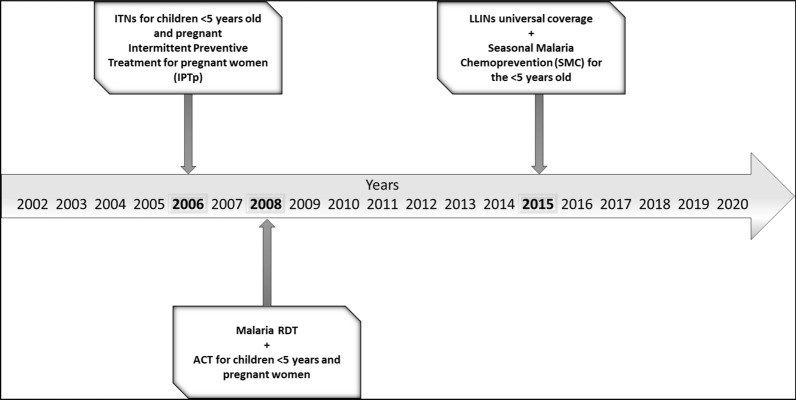
Timeline of the implementation of ongoing malaria control strategies in Dangassa over the past 2 decades. Source: Mali NMPC: National Malaria Control Program (Not published)

This cohort study was performed in the village of Dangassa, located in a high malaria-transmission area of Mali (Sudanese-Guinean zone) from 2012 to 2020. Dangassa is one of the sites of the International Centre for Excellence in Malaria Research in West Africa (ICEMR-WAF) [9] defined as an intense malaria transmission area. Malaria control and prevention strategies in Dangassa are based on case management at community health centres, using rapid diagnostic tests (RDT), arteminisin-based combination therapy (ACT) and ITNs, beginning in 2008. In 2015, the first mass distribution of long-lasting insecticidal nets (LLINs) occurred in the village, achieving universal coverage (one LLIN per every two people). In the same year, SMC was first implemented for children under 5 years during the high-risk season of malaria, from July to October (see Fig. 1).

The aim of the study was to perform a year- and age-specific analysis of the change in malaria epidemiology in human and transmission indicators among malaria vectors in relation to the implementation of control and prevention strategies in the area.

## Methods

### Study site

**Fig. 2:**
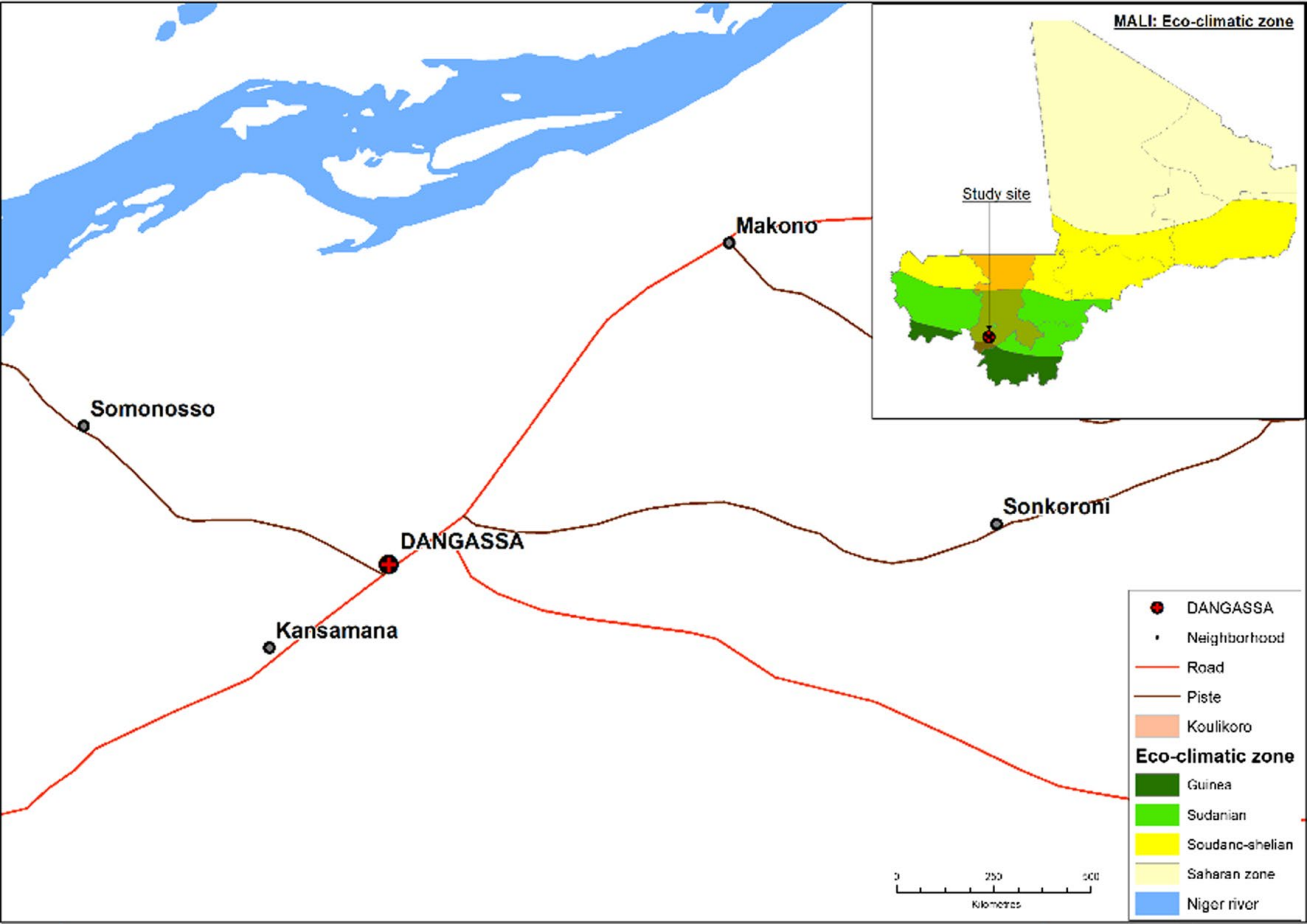
Map of the study site

The study was carried out in the village of Dangassa, Mali, which is situated in the Sudanese-Guinean zone, where malaria transmission can persist for up to 6 months a year. Dangassa village is one of the health zones within the health district of Ouelessebougou, in the region of Koulikoro, Mali (Fig. 2). With approximately 7000 inhabitants, the village is located near the Niger River, approximately 80 km southwest of Bamako. The average annual temperature and rainfall are 27.5 °C and 855 mm, respectively [10, 11]. Malaria transmission is highly seasonal, with more than 65% of cases observed between June and November each year [11]. Dangassa has a community health centre covering 11 surrounding villages and 47 community health workers. In Dangassa, ACT, RDTs, IPTp, and ITNs have been available since 2006–2008, while LLINs universal coverage and SMC for children under 5 years old were introduced in 2015 (Fig. 1) [12].

### Study design

The study was carried out from 2012 to 2020. Before beginning the study, the goals and procedures were presented to the Chief and Elders of Dangassa, to garner community support for the project. After obtaining approval, a general meeting with the community was held, and individual presentations were made to the occupants of candidate houses according to the random selection of households as described below.

Once those communities with high, intermediate and low intensities of *Plasmodium falciparum* infection and disease had been identified by reviewing historical data on the frequency of infection and disease, a census was performed in each candidate community. This approach was in order to identify occupied houses in the community and update the number and ages of the persons in the houses. After all the currently occupied houses in Dangassa had been identified from the updated census, households were randomly selected from the database to obtain a total of 1401 participants of all age groups. Members of selected households were informed about the study and asked if they would consider participating in bi-annual smear surveys.

### Bi-annual smear surveys for the prevalence of *Plasmodium falciparum* infection

Each year, two smear surveys were performed at the beginning (June–July) and the end of the malaria transmission season (October–November). Each survey was done over an average of 12 days at the community health centre of Dangassa. Selected household members were screened for malaria parasitaemia and a structured questionnaire was administered. Information on the history of fever was collected and a clinical examination was performed. Only participants with fever or reported fever within the past 48 h were tested using malaria RDTs. The SD BIOLINE Malaria Ag P.f/Pan test, a qualitative and differential test for the detection of histidine-rich protein II (HRP-II) antigen of *P. falciparum* and common *Plasmodium* lactate dehydrogenase (pLDH) of *Plasmodium* species in whole human blood, was used.

Prevalence of *P. falciparum* infection and parasite density were measured by microscopy during cross-sectional surveys. Blood films were performed (capillary blood) for all participants, regardless of symptoms, to determine asymptomatic malaria parasitaemia. Thick and thin blood film slides were prepared using 10% Giemsa solution for 30 min. The stained slides were then examined under a light microscope, using 100 ×  oil immersion, by an experienced technician. Parasitaemia was calculated per 200 white blood cells (WBC), assuming 8000 WBC/µl of blood [13]. Anaemia was assessed for each participant using HemoCue^®^ Hb 301 System (HemoCue America, Danaher Company, Brea, CA, USA).

Only symptomatic malaria cases, defined as a patient with fever or history of fever within the past 48 h and RDT positive, were treated, free of charge, according to the national policy for malaria case management by the Malian National Malaria Control Programme (NMCP). Slide reading was performed, and records on infection status, malaria parasite species and count of asexual and sexual forms for *P. falciparum* were collected.

### Continuous surveillance for uncomplicated falciparum malaria

During the enrolment process, participants were asked to visit the clinician for any symptoms on any day of the week. Passive case detection of malaria incidence was implemented at the Dangassa community health centre in January 2013. A physician and a medical student were based in the village and allocated a workspace at the community health centre to perform the daily passive case detection year-round. A weekly household visit was performed in randomly selected households to ensure that all malaria cases were seen by the research team. Once a patient visited the health centre, a questionnaire was administered, followed by clinical examination and blood sample collection. Both malaria RDT and finger-prick blood samples, obtained under sterile conditions using disposable equipment, were systematically performed for patients. Parasitaemia was calculated per 200 WBC assuming 8000 WBC/µl of blood [13]. A clinical report and laboratory analysis form for malaria RDT, slide reading, and an anaemia assessment was completed for each visit. Malaria treatment was given based on the malaria RDT result, according to the national malaria control policy by the NMCP.

### Mosquito collection and sample processing

The collection of anopheline mosquitoes was performed by pyrethrum spray catches at the beginning and end of the rainy season 2 weeks before the smear surveys. Forty-five compounds out of the 250 in Dangassa village were randomly selected for mosquito collection. In each selected compound, one room was sprayed. A compound was defined as a group of households in which members share the same meal.

Each survey lasted 12 days during which 15 rooms were sprayed every three days, three times between 07:00 and 09:00 h for indoor resting mosquitoes collection using the Premium Insect Killer, composed of 1.2% dichlorvos, 0.4% fenitrothion and 0.15% tetramethrin.

*Anopheles gambiae *sensu lato (s.l.) specimens were identified morphologically [14] and placed in labelled vials containing 80% ethanol and transported to the laboratory for analysis and species identification. *Anopheles gambiae *s.l. infection rate (IR) and human blood index (HBI) were established using the enzyme-linked immunosorbent assay (ELISA) techniques [15, 16].

### Calculation of prevalence and incidence

The prevalence of malaria was defined as the number of participants with microscopic positive blood smear to sexual and/or asexual *P. falciparum* divided by the number of participants seen at each visit. Incidence rate of clinical malaria defined as history of fever or a measured temperature  ≥ 37.5 °C and a microscopic positive blood smear to sexual and/or asexual *P. falciparum* was defined as the total number of incident malaria cases divided by the total person-time observed in the cohort.

### Statistical analysis

Results were expressed as frequencies and percentages or mean and standard deviations as appropriate. Data were entered in StudyTRAX database management system (version v3.2.0802, StudyTRAX, Macon, GA, USA). The clinical data were analysed in R-studio version 1.3.1093 and GraphPad Prism v.7 Software for Windows [17, 18]. The prevalence of malaria parasitaemia was defined as the proportion of subjects with microscopic *P. falciparum* positive smear. From the surveillance data at the community health center: Passive Case Detection (PCD) among cohort participants, the malaria incidence rate was estimated as the number of new malaria cases per person-weeks during the follow-up period (expressed per 1000 person-weeks and 100 person-weeks, respectively). The following entomological parameters were calculated: vector density per room, human biting rate (HBR), IR, EIR, and HBI. The density of malaria vectors was calculated as the average number of indoor resting mosquitoes per room per day; the HBR was the average number of mosquito bites received by a sleeping person per time unit (blood-fed and half gravid mosquito/number of sleeping people in the room); the IR corresponds to the proportion of *An. gambiae* s.l. carrying *P. falciparum* sporozoites; the HBI was the proportion of female mosquitoes having human blood in their guts. All analyses were carried out with a 5% type I error threshold.

### Ethical considerations

Ethical approvals were obtained from the National Institutes of Health (NIAID), from both Institutional Review Boards (IRBs) of Tulane University (FWA00002055) and the University of Sciences, Techniques and Technology of Bamako in Mali (2011/77/FMPOS). Before patients were enrolled in this study in 2012 and for those enrolled after, a written informed consent was obtained from each participant or their parent/legal guardian if under 18 years old. Please note that the cohort study protocol has been reviewed and renewed annually since that time. Individual informed consent forms were obtained for mosquito collection by Pyrethrum Spray Catches (PSCs) and Human Landing Catches (HLCs) for both room owners and data collectors before starting mosquito collections.

## Results

A total of 1401 children and adults were enrolled to the study in September 2012 with a ratio of 1.18 towards females. The median age was 11 years (maximum 86 years old). Adults aged over 20 years represented 33% of the study population, while children under 5 years represented 18% of the study population and children aged 5–9 years represented 19% of the study population (Table 1).

**Table 1:** Sociodemographic description of the study population

Characteristic	Screening and enrollment 2012 N = 1401
Sex	
Male	642 (46%)
Female	759 (54%)
Median Age (min, max)	11 (0, 86)
Age groups	
< 5 years	249 (18%)
5–9 years	273 (19%)
10–14 years	258 (18%)
15–20 years	156 (11%)
> 20 years	465 (34%)

The study participants were enrolled in 2012. However, the malaria passive case detection at the community health centre started in January 2013 while the first cross-sectional survey was performed in 2013. Due to funding shortage in 2017, no cross-sectional survey could be performed while malaria passive case detection (PCD) was ongoing.

**Fig. 3:**
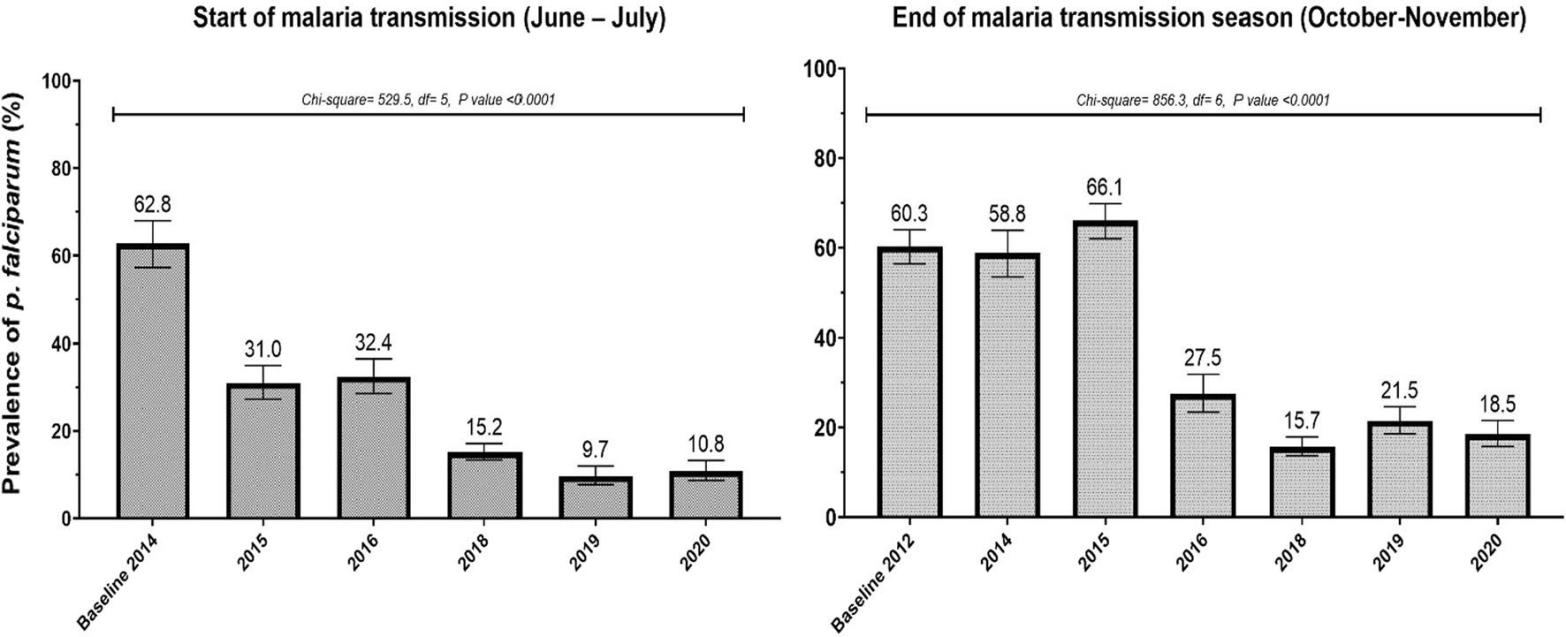
Overall seasonal variations of *P. falciparum* infection prevalence at the start and end of malaria transmission from 2012 to 2020

The baseline survey occurred in October 2012 (end of the transmission season) while the first survey at the start of the transmission season occurred in June 2014. Therefore, for comparison and estimation of the change in *P. falciparum* infection, data collected in June 2014 was used as the baseline for the start of the transmission season and October 2012 as the baseline for the end of the malaria transmission (Fig. 3).

The mean prevalence of *P. falciparum* parasitaemia at the start of the malaria transmission (June-July) shows a significant year-to-year variation from 2014 to 2020. *Plasmodium falciparum* infection prevalence varied from 62.8% in June 2014 to 10.8% in June 2020 with an overall decrease of 84.5% (X^2^  = 529.5; p  < 0.0001).

*Plasmodium falciparum* infection at the end of the malaria transmission season (October–November) also showed a significant variation from 2012 to 2010 with 60.3% and 18.5% in 2012–2020. The estimated decrease was approximately 65.8% (X^2^  = 856.3; p  < 0.0001) (Fig. 3).

**Fig. 4:**
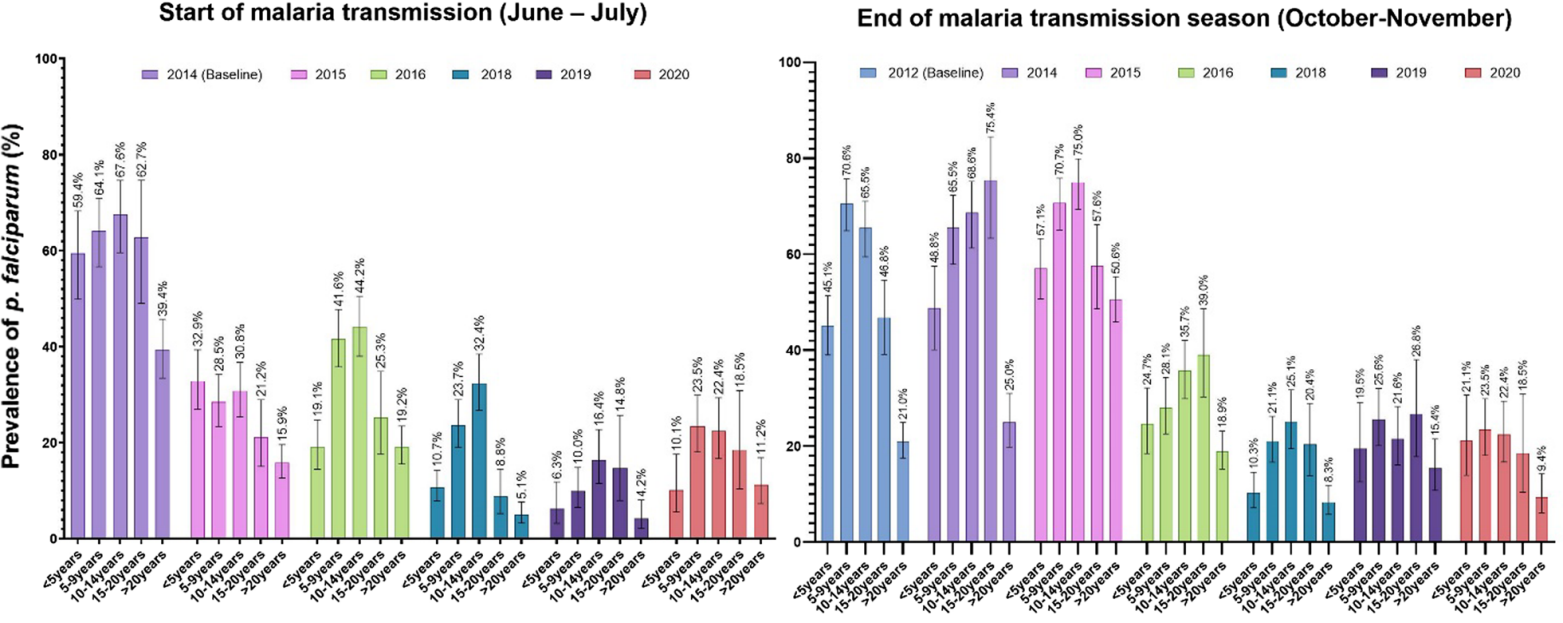
Age-specific *P. falciparum* malaria infection (number of smear positive/age group population size within the cohort) at the start and the end of the malaria transmission season from 2012 to 2020. Data from 2013 and 2017 are missing

Figure 4 provides age-specific malaria infection rates per year, with August 2012 as the baseline for comparison. At the start of the malaria transmission season, a significant decrease in asymptomatic carriage of the malaria parasite was observed over multiple years, among all age groups. Parasitaemia was much higher among children under 5 years (33–59.4%) compared to other age groups. However, in 2016 an age-shift of infection prevalence was observed, with more children aged 5–9 years and 10–14 years infected than all other age groups. A similar trend was observed through 2020. The year-to-year decrease in infection prevalence was observed among both age groups during the study period. However, this change was more significant among children under 5 years, with a decrease of 49.3% (95% CI 37.2–59.2) vs 43% (95% CI 35.7–49.6) between 2014 and 2020.

Compared to the start of the malaria transmission season, from the beginning of the study in 2012 to the end of the study in 2020, parasitaemia at the end of the malaria transmission was always higher among children aged 5–14 years compared to the under 5 years age group with an average parasitaemia of 44.2 and 32.4%, respectively. Comparison of *P. falciparum* infection prevalence at the peak malaria transmission season from 2012 to 2020 shows 24% (95% CI 12.7–33.5) and 45% (95% CI 38.9–50.7) decreases among children under 5 years and children 5–14 years, respectively.

**Fig. 5:**
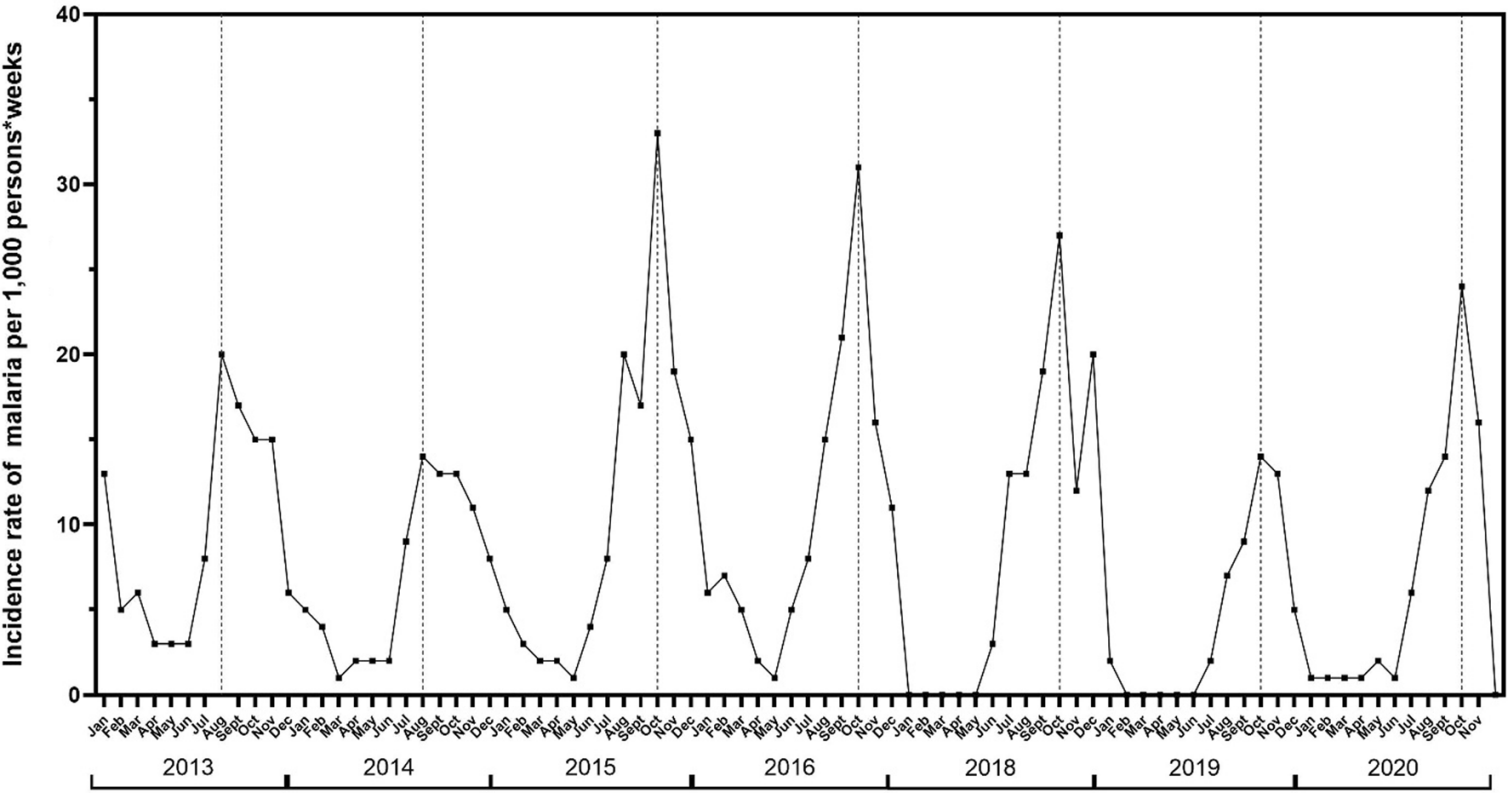
Overall seasonal variations of malaria monthly incidence rate from 2013 to 2020

Incidence of symptomatic malaria is shown in Fig. 5. This Figure shows the seasonality of malaria, with incidence rates lower than 5% during the dry season, from January to June, and closer to 10% in July, except in 2018 where incidence rate was closer 15 case per 1000 person-weeks. Each year about 80–90% of all malaria cases were observed from July to November in Dangassa. The temporal trends of malaria occurrence were similar for both years, with two peaks observed in 2015 and 2016 with 34 and 32 cases per 1000 person-weeks, respectively (Fig. 5).

**Fig. 6:**
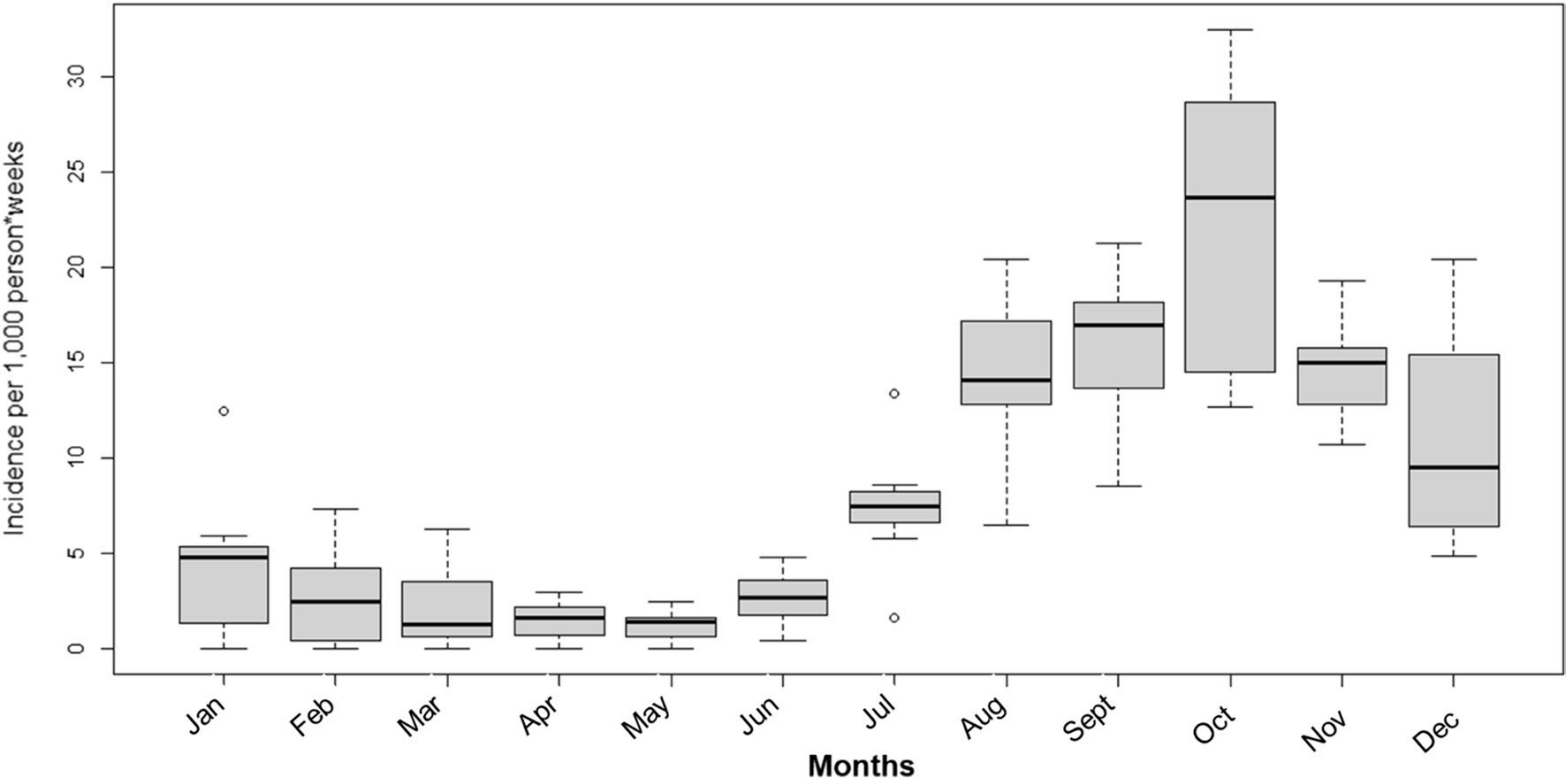
Mean monthly variation of malaria incidence rates per 1000 person-weeks in Dangassa from 2013 to 2020

Time-series analysis approach was used to estimate each month the variation of malaria incidence per 1000 person-weeks from 2013 to 2020. Data show that malaria is highly seasonal in Dangassa with fewer than 5 cases per 1000 person-weeks from January to June each year corresponding to the dry season. A fluctuation of malaria cases started from July (7.5 malaria cases per 1000 person-weeks), peaked in October with approximately 22.5 malaria cases per 1000 person-weeks, and then decreased progressively to 9 malaria cases per 1000 person-weeks (Fig. 6).

**Fig. 7:**
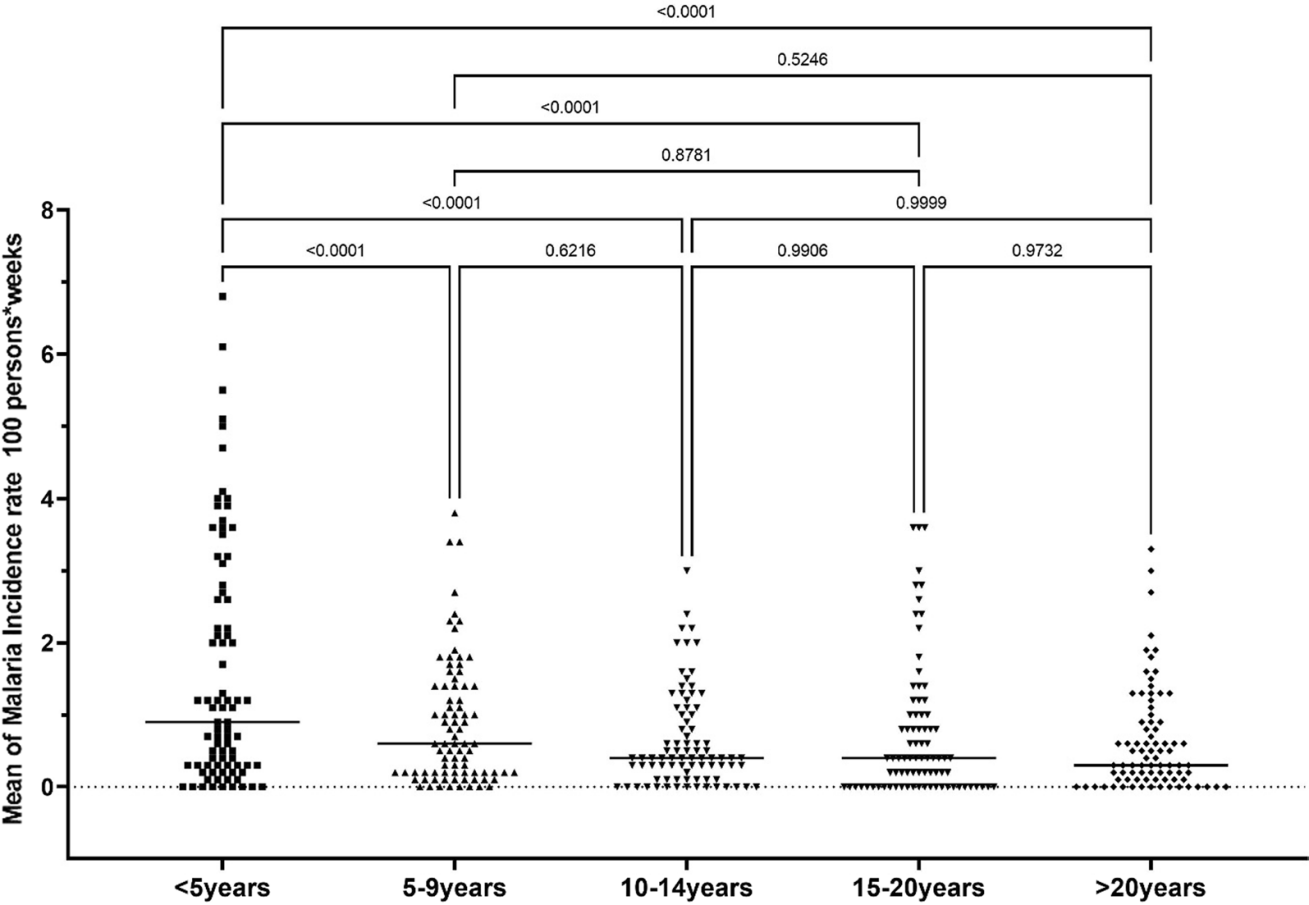
Age-specific mean of malaria incidence rate from 2013 to 2020. Average incidence rate is estimated for each group. Each dot represents the yearly incidence rate per month. The lateral bar represents average incidence rate variation per month for each age group during. One-way ANOVA by Tukey’s multiple comparison test with a significance rate of 5% was used to compare the difference observed between means

In Fig. 7, mean incidence rate is significantly higher among the children under 5 years compared to any other age group during that period (p  < 0.05). However, the other age groups did not show any statistically significant difference during the study period, in terms of incidence rates (p  > 0.05).

### Density and human blood index of anopheline mosquitoes

**Fig. 8:**
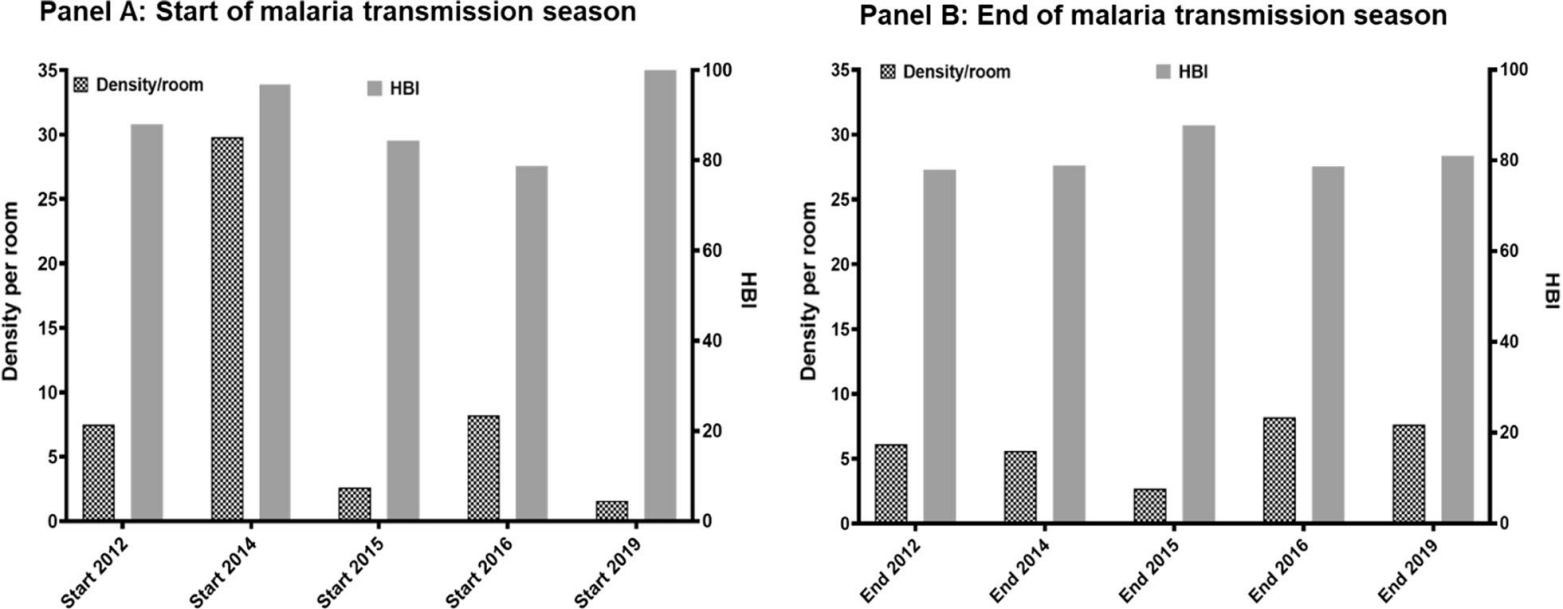
Monthly mean density (MMD) and human blood index (HBI) of *An. gambiae* s.l. in human dwellings in Dangassa

The mean density at the start of the malaria transmission season shows a significant year-to-year variation (from 2 *An. gambiae* s.l. per room in 2019 to 30 *An. gambiae* s.l. per room in 2014) while the HBI remains extremely high regardless of the year (80–100%). Regardless of the year, density of mosquitoes at the end of the malaria transmission season was less than 25 mosquitoes per room). However, the HBI remained as high as at the start of the malaria transmission season, varying from 78 to 90% (Fig. 8).

### Mean human biting rate, infection rate and entomological inoculation rates in *Anopheles gambiae* s.l.

**Fig. 9:**
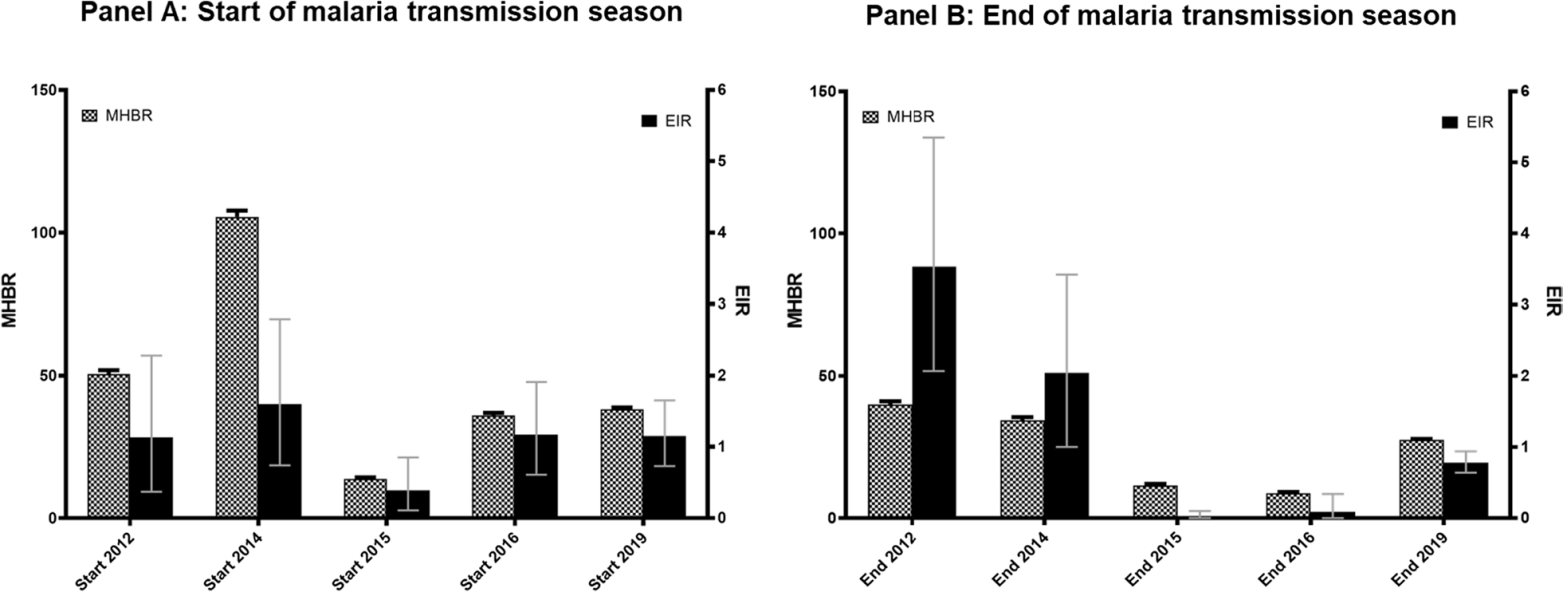
*Anopheles gambiae* s.l. monthly biting rates and Entomological Inoculation Rate (EIR) variation of malaria transmission from 2012 to 2019. *MHBR* mean human biting rate. *EIR* entomological inoculation rate

Figure 9 shows the mean monthly human biting rate (MHBR) and the EIR from 2012 to 2019. At the start of the malaria transmission, the highest MHBR were observed in June 2014 with approximately 110 bites/person/month, followed by a four times decrease at the same moment of the year in 2015. MHBR was always lower at the end of the malaria transmission with the highest MHBR observed in 2012 and 2014 with 45 and 43.5, respectively while at the start of the malaria transmission, the highest MHBR was observed in year 2014 with 105.5 bits/person/month (Fig. 9). EIR at the start of the malaria transmission season varied from 1.1 to 1.5 infective bites/person/month except in 2015 where EIR was less than 0.5 infective bite/person/month). However, in contrast to MHBR, EIR was significantly high at the end of malaria transmission varying from 0.01 to 4.02 infective bites/person/month in 2016 and 2012, respectively. Overall, a significant decrease was observed in both MHBR and EIR from 2015 to 2019 (p  < 0.05).

## Discussion

### Prevalence of asymptomatic malaria in the study population

The study assesses *P. falciparum* infection prevalence in Dangassa over 8 years, at the start and end of the transmission season, with all age groups represented. High malaria prevalence was observed at baselines (end of malaria transmission season in 2012 and start of the malaria transmission season in 2014) with 60.3 and 62.8% of participants having positive blood smears, respectively. A significant decrease was observed in 2015, with a 50% drop at the start of the transmission season. However, in the same year prevalence at the end of the transmission season was similar to that observed in the previous year in the same month. Since 2016, a year-to-year decrease in prevalence of asymptomatic malaria was observed at both start and end of the transmission season with the lowest prevalence in October 2018 (15.7%) and June 2019 (9.7%). This decrease can be related to the universal ITN coverage campaign in 2015 followed by the efficient implementation of SMC in 2016 in the study area. It is worth mentioning that observations occurring in a single year may be due to random fluctuations, and those observations stable over a 2-year interval are consistent with true patterns.

Data show the presence of relevant reservoirs for malaria parasites in the study population, which can contribute to maintaining the transmission immediately after the onset of the rainy season and the presence of malaria vectors in the study area. A similar study in Bandiagara, Mali shows that asymptomatic carriage persisted in the community, with a ratio to clinical episodes from high to low transmission periods of about 0.5–5 episodes per child. Throughout the entire dry season, malaria transmission was low, and asymptomatic carriers were the only reservoir of parasites during this time [19]. The decrease in asymptomatic carriage of malaria parasites in 2016 can be explained by the combined effect of universal coverage of LLINs in 2015, followed by the countrywide implementation of SMC for children under 5 years in 2016 [11].

Seasonal variations of age-specific carriage of malaria parasites show that children aged 5–9 years and 10–14 years represent the main reservoir for parasites in the study area, at both the start and end of the rainy season. Similar patterns have been described in Sélingué, Mali with a shift of both asymptomatic and symptomatic malaria prevalence and incidence among older children, who are unfortunately not yet targeted by specific malaria interventions, such as free testing and ACT, or SMC [20]. The high infection rates observed at the start of the malaria transmission season suggest adoption of specific strategies, such as mass drug administration or SMC for older children, starting a month or two before the rainy season in Dangassa, could help reduce malaria incidence in the region [11, 19, 21].

### Malaria seasonality and age-specific mean incidence of malaria in Dangassa

Symptomatic malaria is highly seasonal in Dangassa, with a peak observed in August during the first 2 years of the study (2013 and 2014) and in October 2015 and 2020. Relatively low incidence was observed during the dry season from January to June each year. This pattern has been described in several regions of Mali [19, 20]. However, the length of the transmission season in Dangassa is much longer than the 4 months described in many other regions of the country, calling for a reinforcement and extension of the duration of control interventions, such as increasing SMC from 4 to 5 months (July–November instead of July–October). In addition, mass administration of malaria drugs at the end of the dry season can contribute to the decline of clinical cases during the transmission season [22, 23].

Compared to the high prevalence of asymptomatic malaria among older children over the follow-up period, incidence of malaria was higher among children under 5 years, regardless of their benefit of all present interventions in the study area. These observations show that malaria disease is more significant among this group than the rest of the population. Several hypotheses can explain this situation: (1) appropriate treatment with ACT given free of charge to children under 5 years may lead to the lowest asymptomatic carriage of the parasites observed; (2) compliance to SMC treatment (proportion of children receiving all three doses of the treatment each month) may be overestimated, since only the first dose is taken under direct observation and parents are asked to administer the second and third doses at home; (3) experiencing many clinical episodes at an early age is known to induce premonition for the 5 years and older age group, leading to a decrease in the risk of presenting with symptoms in cases of low parasitaemia [24, 25].

### Malaria vector population: density, human biting index and entomological inoculation rate

The main malaria vector in Dangassa is *An. gambiae* s.l. representing more than 90% of the *Anopheles* fauna, regardless of the season. Similar results have been reported by several authors in localities near Dangassa [26, 27]. The study results show that *An. gambiae* s.l. density per room varied significantly by month, season and year. These fluctuations are related to the rain-dependency of the breeding sites of *An. gambiae* s.l. [28, 29].

*Anopheles gambiae* s.l. was very anthropophilic. Outdoor watching of television and/or other domestic activities may have allowed mosquitoes access to human blood sources despite LLIN universal coverage. In addition, outdoor resting behaviour developed by malaria vectors in this area may compromise the efficacy of indoor-based interventions, such LLINs [30].

As expected in seasonal malaria transmission areas, this study’s results show higher EIRs at the end of the rainy season compared to the beginning [26, 30–32]. There was a trend of decrease in both MHBR and EIR observed from 2015 to 2020 (with some fluctuation at the onset of the rainy season in 2018 and 2020), which can be related to the increase in malaria control intervention as described elsewhere [33, 34]. The findings are consistent with observation made in Mopti region of Mali from 1999 to 2006 after a large deployment of ITNs [35].

## Limitations

The work focused on a single rural site in Mali which may not represent suburban or urban areas. In 2013 and 2017, prevalence studies could not be carried out due to budgetary shortage causing missing data. Passive case detection data are also missing for 2017 corresponding to the time between two rounds of ICEMR. Entomological data in 2020 are not presented because sample processing was not possible because of shortage of laboratory supplies as a consequence of the Covid19 pandemic.

## Conclusions

Despite the decrease observed both in asymptomatic and symptomatic malaria since 2015, malaria transmission remains hyperendemic in Dangassa. Older children not targeted by many of the interventions seem to be more affected and represent the main malaria parasite reservoir in this community. High EIRs observed can explain the rapid increase of malaria cases at the onset of rainy season in Dangassa. Updated strategies such as extension of free diagnostics and treatment and SMC to older children, malaria mass drug administration before the start of the transmission season and IRS could be effective in reducing the malaria burden in Dangassa.

## Data Availability

The datasets generated and/or analysed during the current study are available from the corresponding author on reasonable request. We are in process to made data sets accessible in the next months on https://clinepidb.org/ for public access.
